# Optofluidic photonic crystal fiber platform for sensitive and reliable fluorescence based biosensing

**DOI:** 10.1364/BOE.527248

**Published:** 2024-06-13

**Authors:** Baptiste Moeglen-Paget, Jayakumar Perumal, Georges Humbert, Malini Olivo, U. S. Dinish

**Affiliations:** 1 Xlim Research Institute, UMR 7252, CNRS, Université de Limoges, 123 Avenue Albert Thomas, 87000 Limoges, France; 2 A*STAR Skin Research Labs (A*SRL), Agency for Science, Technology and Research (A*STAR), 31 Biopolis Way, #07-01 Nanos, Singapore 138669, Republic of Singapore; 3 georges.humbert@xlim.fr; 4 malini_olivo@asrl.a-star.edu.sg; 5 dinish@asrl.a-star.edu.sg

## Abstract

Biosensing plays a pivotal role in various scientific domains, offering significant contributions to medical diagnostics, environmental monitoring, and biotechnology. Fluorescence biosensing relies on the fluorescence emission from labelled biomolecules to enable sensitive and selective identification and quantification of specific biological targets in various samples. Photonic crystal fibers (PCFs) have led to the development of optofluidic fibers enabling efficient light-liquid interaction within small liquid volume. Herein, we present the development of a user-friendly optofluidic-fiber platform with simple hardware requirements for sensitive and reliable fluorescence biosensing with high measurement repeatability. We demonstrate a sensitivity improvement of the fluorescence emission up to 17 times compared to standard cuvette measurement, with a limit of detection of Cy5 fluorophore as low as 100 pM. The improvement in measurement repeatability is exploited for detecting haptoglobin protein, a relevant biomarker to diagnose several diseases, by using commercially available Cy5 labelled antibodies. The study aims to showcase an optofluidic platform leveraging the benefits provided by optofluidic fibers, which encompass easy light injection, robustness, and high sensitivity.

## 1. Introduction

Biosensing holds a prominent role in modern scientific research, driven by its significant influence across diverse applications, including medical diagnostics, environmental monitoring, and biotechnology. At the heart of biosensing, biosensor platforms stand as indispensable tools, enabling selective detection of biological molecules. With an increasing demand for high sensitivity, real-time monitoring, and adaptability to different molecules, novel technologies and platforms are continually emerging. While some platform such as Western blot or enzyme-linked immunosorbent assay (ELISA) has shown to be robust for biomarker detection, particularly proteins, it is not without limitations [1–4]. These methods involve multi-steps protocol, require prolonged operation times and expertise, suffer from non-specific detection and demand specific equipment available only in selected laboratories. New optical methods, such as waveguides or optical fibers, provide a solution to the constraints encountered by devices employing focused laser beams through microscope objectives for fluorescence biodetection.

Photonic crystal fibers (PCF) are optical fibers made of a periodic microstructure of air holes running along the fiber length. They have led to the development of optofluidic fibers where liquid can be injected into large air holes allowing efficient light-matter interaction through evanescent wave or through direct interaction in the case of hollow-core fibers. In fluorescence sensing, these fibers have shown to be an ideal optofluidic platform due to the small volume of samples that can interact with the light while offering higher sensitivity and repeatability [5–8]. This opens new avenue for real-time monitoring, multiplexing or also label free detection, which are essential factors in the case of biomarker detection [9–13]. Different demonstration for biodetection with Raman spectroscopy, refractive index sensing or fluorescence spectroscopy have been shown using principally two design of PCF fibers which are solid-core or hollow-core PCF [14,15].

Suspended-Core PCF (Sus-PCF) is a special kind of PCF composed of a solid core surrounded by 3 large holes. This simple design allows the fabrication of very small core fiber (few micrometer diameter) for favoring light-matter interaction through the evanescent field [16–22]. The portion of the light propagated outside the core increases with smaller core size leading to stronger interactions with the fluorophores in the vicinity of the core. In this way, sensing can be done along the entire fiber length, leading to an increase of the interaction between the excitation light and the fluorophores. The potential of this configuration has been already shown in various fluorescence sensing applications, such as chemical detections (e.g., Zinc and Aluminium ions) and biosensing (e.g., protein, DNA, antibody detection) [23]. However, these applications have been realized in a laboratory with complex optical setup that are hardly compatible to be used in a clinical environment [24–27]. The development of an optofluidic platform for real-life fluorescence biosensing requires an ease of use, high sensitivity and reliable measurements. One first attempt, to improve the reliability, was the development of an automated alignment setup using an optical feedback to ensure stable optical coupling into the core of a Sus-PCF (D_core_ = 2.1 µm) [28]. However, this approach is relatively complex and require a careful alignment of the fiber.

In this study, we report the development of a new optofluidic PCF platform for sensitive and reliable fluorescence based biosensing. We fabricated Sus-PCFs optimized for fast liquid infiltration into the air-channel, and for improved coupling efficiency of light through the microscope of our setup. We demonstrate the better efficiency of the fiber for detecting fluorescence dye Cyanine 5 in comparison with measurement in a standard cuvette. To highlight the practical significance of the fiber performances in sensitivity and in measurement reliabilities, we demonstrate the immuno-detection of haptoglobin protein through a simplified modality based on the sandwich method. Haptoglobin is a relevant biomarker. It is a glycoprotein found in blood plasma, playing a critical role in diagnosing conditions such as hemolytic anemia, certain cancers, and inflammatory disorders. Reliable quantification of haptoglobin contributes to the early diagnosis and monitoring of conditions where its levels are indicative of underlying health issues [29–32].

## 2. Material and method

### 2.1 Fiber fabrication

Sus-PCF with three different core sizes (2 µm, 3 µm, and 3.5 µm) were fabricated using the conventional stack-and-draw process [33,34]. Initially, three capillaries were drawn and assembled into a tube. Subsequently, this assembled stack underwent drawing to form a preform with a few mm diameter. The preform was then placed within a jacket tube and further drawn to produce the final fibers. To achieve fiber samples with the specified core sizes, precise adjustments were made to the pressure applied in the capillaries, the descent speed of the preform in the furnace, and the drawing speed during the fabrication process. After fine-tuning the fabrication parameters for a particular core diameter, the process enables the drawing of several tens of meters of fiber with constant dimensions (i.e. external diameter variation of about +/1 µm). Moreover, the cross section of the fiber is measured continuously during the fabrication process using a microscope to control the geometry and the fiber dimensions. This approach ensures stability and repeatability, guaranteeing uniform fiber characteristics for all measurements and minimizing design uncertainties.

### 2.2 Light coupling simulation

Numerical simulations using the finite element method in COMSOL were conducted to calculate the coupling efficiency between a Gaussian beam (from the excitation laser) and the fundamental mode of a Sus-PCF, with different core diameters. The excitation wavelength was set at 638 nm, corresponding to the central wavelength of the laser. Knowing the initial beam diameter at the output of the laser, the numerical aperture, and the focal length of the microscope objective used for light coupling into the fiber, we calculated that the size of the laser beam, at the waist position, is about 2.14 µm diameter. Alignment uncertainties from an operator are simulated by computing the coupling efficiency for 200 random beams centred within a circle of 1 µm around the Sus-PCF core. The maximum of coupling efficiency is obtained in the case of perfect alignment between the centre of the fiber and the laser beam. However, a beam not centred with the core correspond to the case where alignment has been done with some manual error from the operator. The impact of the misalignment on the coupling efficiency are computed by taking the average value among 200 random point.

After calculating the electromagnetic field distribution of the Gaussian beam and of the fundamental mode of the fiber, the normalized coupling coefficient (C) was derived from the continuity of the transverse field components across the fiber's cross-section. This calculation has been done using the following Equation 1 [35].

$$C=\frac{1}{4}\times \frac{\int \left({\vec{E}}_{\;in}^{*}\left(x,y\right)\times \vec{H}\;\left(x,y\right)+\vec{E}\left(x,y\right)\times {\vec{H}}_{in}^{*}\left(x,y\right)\right)dxdy}{\sqrt{\frac{1}{2}\int Re(\;{\vec{E}}_{\;in}\left(x,y\right)\times {\vec{H}}_{\;in}^{*}\left(x,y\right))dxdy}\times \;\sqrt{\frac{1}{2}\int Re(\vec{E}\left(x,y\right)\times {\vec{H}}^{*}\left(x,y\right))dxdy}}$$

with E_(x,y)_ and H_(x,y)_ are the x and y components of the electric and magnetic fields of the fundamental mode guided inside the fiber. E_(x,y in)_ and H_(x,y in)_ are the x and y components of the electric and magnetic fields of the laser Gaussian beam.

### 2.3 Chemicals

(3-Aminopropyl) triethoxysilane (APTES, 440140) was bought from Sigma Aldrich. Bovine Serum Albumin (BSA, SLCG7366) was obtained from Sigma Aldrich. Haptoglobin protein were acquired from Dako Denmark. N-hydroxysuccinimide (NHS, 24500) and 1-éthyl-3-(3-diméthylaminopropyl) carbodiimide (EDC, 22980) were furnished by Thermo Fisher Scientific. A custom Cy5-labeled antibodies solution were obtained by GenScript. Dimethyl Sulfoxide (DMSO, K43987812) was bought from Merck.

### 2.4 Fluorescence measurement

**Fig. 1.**
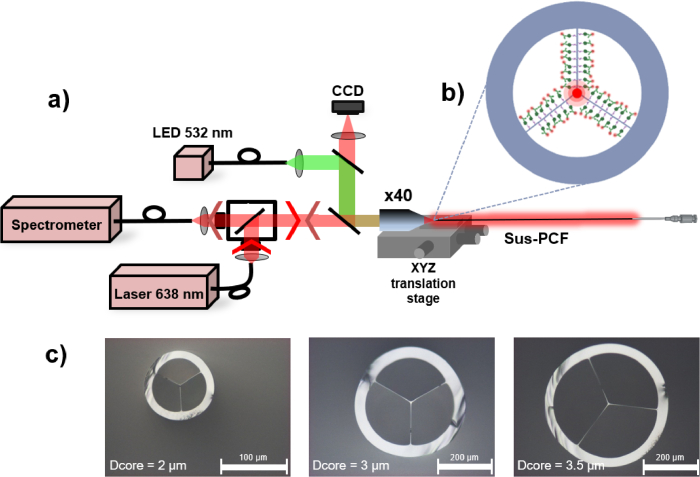
a) Optical setup of the platform for fluorescence measurement inside Sus-PCF with a green LED for alignment and a laser emitting at 638 nm for dyes excitations. The Sus-PCF is mounted on a XYZ translation stage to control the position with a high precision. A microscope objective X40 (Olympus) is used to couple excitation light in the Sus-PCF and also collect the fluorescence emitted inside the fiber holes and propagating back in the fiber core. Moreover, a 3D printed holder can be added to the XYZ stage in order to set the cuvette after the microscope objective. A CCD camera allow to control the light coupling (reflected on the core surface). A set of filters are used to obtain a narrow excitation bandwidth (FF01-638/8-25 from Semrock), then filter out the laser signal and collect only fluorescence (LP02-638RU-25 from Chroma), (BLP01633R-25 from Chroma). b) Cross section of the fiber where the light is coupled in the solid core at the center of the fiber. Evanescent wave propagating in the three holes of the fiber is shown with transparent red and simulate the zone of interaction between the excitation light and the biological target. c) Microscope photographs of the Sus-PCF cross section with a core diameter of 2 µm, 3 µm and 3.5 µm.

As depicted in Fig. 1(a), a laser diode (VD-IV-L-A01, Viasho) emitting at 638 nm with a bandwidth of 8 nm and an adjustable power from 1 to 50 mW is employed to excite the fluorophore inside the fiber. For precise alignment within the Sus-PCF, illustrated in Fig. 1(b), the fiber is positioned on a XYZ translation stage (Nanomax MAX313D/M, Thorlabs). The coupling is controlled using a camera to image the reflected beam on the fiber core (as a microscope). A LED emitting at 532 nm helps in the initial alignment, with the laser turned off to prevent premature excitation of the Cy5 dyes. Furthermore, our setup incorporates three sets of filters. The bandpass filter (with a bandwidth of 2 nm centered at 638 nm, FF01-638/8-25 from Semrock) is used to clean the laser spectrum. Dichroic mirror and long pass filters are used to reflect and to filter the fluorescence signal to the laser (LP02-638RU-25 from Chroma and BLP01633R-25 from Chroma). This setup is designed to work with diverse dyes such as Cyanine 5 or Alexa Fluor 647, which have peak excitation at 650 nm and emissions at 670 nm. The fluorescence signal back-propagated in the fiber core is measured by a spectrometer (Maya 2000, Ocean Optic). This configuration has been demonstrated to be more efficient and convenient as the signal intensity is not dependent on the fiber length optimization (i.e. the optimization of the fluorescence signal intensity versus its absorption by the fiber attenuation) [26]. Furthermore, the experimental setup is simplified to only one microscope objective on one side of the fiber and also opens up the possibility to keep the other fiber’s end attached to a syringe needle in order to filtrate and sense different liquids.

A numerical interface, done with MATLAB, enables the real-time monitoring of fluorescence intensity, allowing observation of potential changes due to dye photobleaching.

A needle is attached at the end of the fiber for enabling fast and easy liquid infiltration into the air-holes by simply connecting it with a syringe. A push-syringe is used to control the volume and the speed of injection. All fiber lengths were standardized to 17 cm. Fluorescence measurement of Cy5 dyes diluted in DMSO solvent was conducted using a standard cuvette, by replacing the fiber by the cuvette. In this case, the cuvette was placed in front of the objective lens. A 3D printed holder has been fabricated to fit the mechanical stage and to control the position of the cuvette in order to have the same excitation condition of the dyes and to be able to compare the fluorescence intensity.

### 2.5 Protein detection

**Fig. 2.**
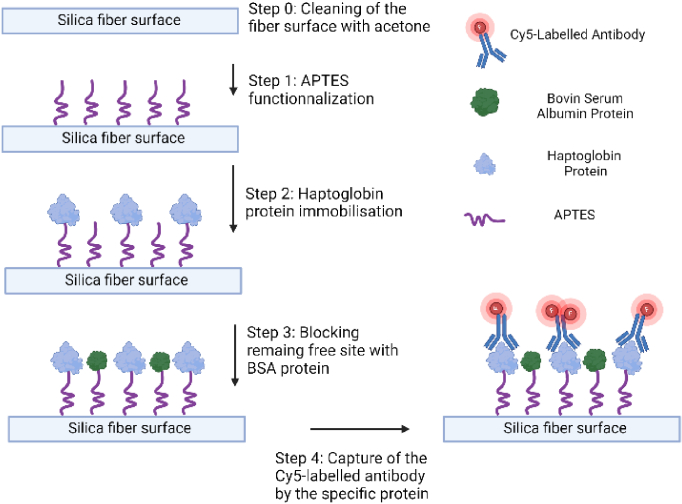
Immobilisation protocol of Cy5 labelled antibody for specific detection of Haptoglobin protein.

Before initiating the protocol, the fiber is washed with acetone to eliminate all impurities and dust. Between each step, the fiber is washed by pumping air and buffer at a rate of 100 µL/min. In the subsequent injections, we maintain a rate of 50 µL/min, and a sample volume of 0.3 mL is introduced to facilitate effective functionalization of the fiber surface and binding of the antibody, similar to an incubation period in the case of planar substrate. First, a 2% solution of APTES in acetone is employed to create an initial layer of amino groups (NH_2_) for the haptoglobin protein immobilization. To achieve this, a mix of EDC at 10 mM and, NHS at 25 mM solution is injected in the fiber to activate the carboxyl groups on the fiber surface. Various concentrations of haptoglobin diluted in buffer are subsequently introduced and captured at the fiber surface through the EDC/NHS immobilization protocol [36,37]. Additionally, a 2% BSA in water is used to block any remaining active NH_2_ group, preventing non-specific binding of antibodies directly with the APTES. Finally, Cy5-labeled haptoglobin antibodies (Cy5-Hb-Ab) at 10 µg/mL are injected into the fiber and captured by the haptoglobin protein. For this step, the rate of injection is set to 20 µL/min. After few rounds of washing, with water and air, to remove unbound Cy5-Hb-Ab, resultant fluorescence spectra measurement corresponds to only the antibodies that has been immobilized with the haptoglobin protein. The detailed protocol is illustrated in Fig. 2. For control sample, only BSA protein were immobilized inside the fiber prior to flowing Cy5-Hb-Ab. In this case, Cy5-Hb-Ab should not attach to the BSA protein (nonspecific) and as a result, fluorescence signal will be very weak or negligible. Finally, the fiber is aligned with the laser and the spectrometer for measurement. The evanescent part of the propagated light will then interact with the Cy5-Hb-Ab attached at the inner surface of the fiber core. As the laser excitation and the fluorescence signal are mainly guided inside the core, they are weakly attenuated by the liquid absorption (in contrast to cuvette measurements) allowing dyes excitation along all the fiber length.

## 3. Results and discussion

### 3.1 Sensitivity

**Fig. 3.**
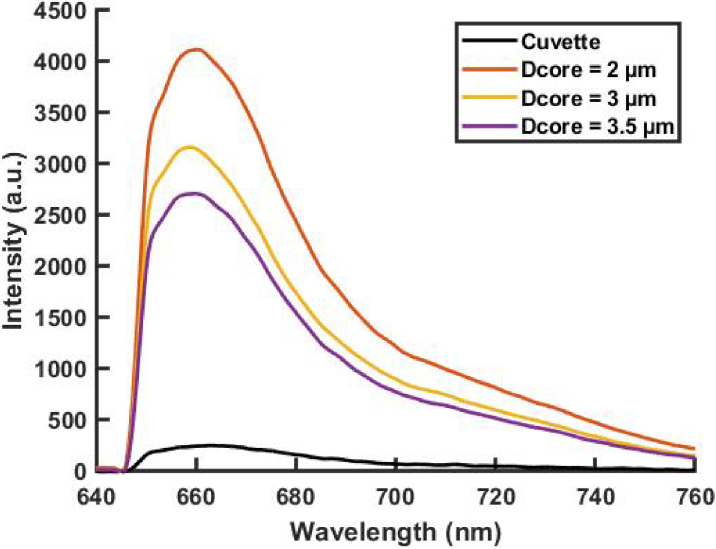
Fluorescence spectra of Cy5 dye (c = 10 nM) measured in cuvette (L = 1 cm) and in Sus-PCF with different core size (2 µm, 3 µm and 3.5 µm, fiber length = 17 cm, laser power 0.5 mW). Each spectrum is an average value of 8 measurements done for each fiber.

Figure 3 presents the measured spectra of Cyanine 5 dye, in cuvette alongside an average of eight spectra obtained for the three Sus-PCF with different core sizes (2 µm, 3 µm, 3.5 µm). Measurements conducted within the fiber exhibit a notably higher fluorescence signal with respectively 4100, 3300 and 2700 counts for 2 µm, 3 µm, and 3.5 µm respectively compared to 250 counts for the cuvette. This enhancement attributed to the fiber is quantified by calculating the ratio between the number of counts in the fluorescence peak for the three fibers and the cuvette. Sus-PCF with core sizes of 2 µm, 3 µm, and 3.5 µm demonstrated enhancements of 17, 13, and 11 times, respectively compared to the measurement in cuvette. The observed increase in intensity can be rationalized by the extended interaction length offered by the fiber, in comparison to the cuvette. Additionally, 2 µm core fiber exhibits a higher fluorescence intensity than the other fibers, which is due to the larger evanescent field induced by the smaller core and leading to an increase of the amount of light interaction with the dye filled in the fiber holes. The calculated fraction of evanescent field (at 638 nm) is 1.27% and 0.28%, for the Sus-PCF with a core size of 2 µm and 3.5 µm, respectively. It is worth to notice that in comparison to the full (100%) propagation of the light in the liquid (in a cuvette), the portion of the light propagating in the liquid-filled holes of the fiber is rather small. The larger fluorescence signal obtained in the Sus-PCF demonstrates the strong limiting factor of the light absorption by the liquid and the gain offered by the long interaction length of the fiber.

### 3.2 Coupling efficiency

We have investigated experimentally the coupling efficiency for each core size employed in the previous measurements but also with numerical simulation. The measured coupling efficiency is quantified as the ratio between the power measured after the microscope objective and the power measured at the fiber's output. Table 1 details the maximum coupling efficiency achievable for each core diameter, accompanied with images of the light propagated in the fiber core (measured with a camera positioned at the output of the fiber). These images validate a correct propagation of the light into the fiber core.

**Table 1.** Experimental coupling efficiency measured as the ratio between input laser power (3.6 mW) and output power in transmission. For each case an image of the intensity distribution of the light at the fiber output is presented. Scale bar length = 14 µm

Fiber core	2 µm	3 µm	3.5 µm

Coupling efficiency	71%	79%	85%

Light distribution of the fiber output	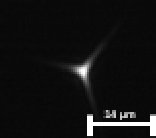	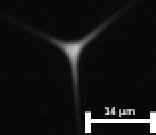	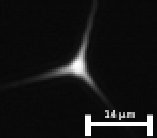

**Fig. 4.**
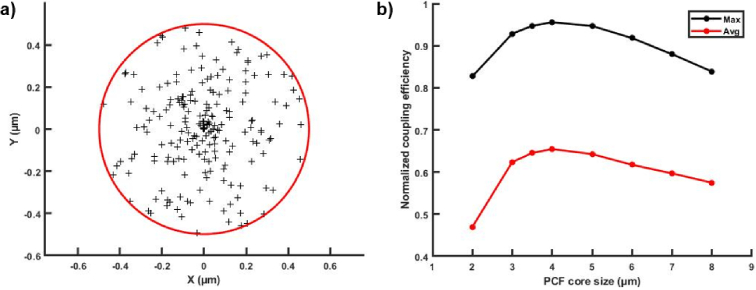
a) Distribution of the laser beam center randomly positioned (200 times) within an area limited by a circle of 1 µm radius around the center of the fiber. b) Maximal and average coupling coefficient values for Sus-PCF with different core diameters.

The measured coupling efficiencies are about 71%, 79%, and 85% for the core size of 2 µm, 3 µm, and 3.5 µm, respectively (Table 1). These values are consistent with the numerical simulations of the coupling efficiency (Fig. 4(b).) that exhibits a maximum for a core size of about 4 µm. This indicates that even if the Sus-PCF with a core size of 3.5 µm leads to a lower signal intensity (than the one obtained with two other fibers), this fiber enables the highest coupling efficiency (i.e. an average coupling of 65% for coupling position randomly positioned within a circle of 1 µm diameter around the core center). Moreover, the ratio between the average and maximum coupling efficiency shows that for a misalignment with a fiber core of 2 µm, the coupling efficiency drops by 53% while in the case of a 3.5 µm fiber core, it only drops by 36%. In other terms, this simulation study shows that using a 3.5 µm core is minimizing the effect of misalignment.

### 3.3 Evaluation of the repeatability and the limit of detection

**Fig. 5.**
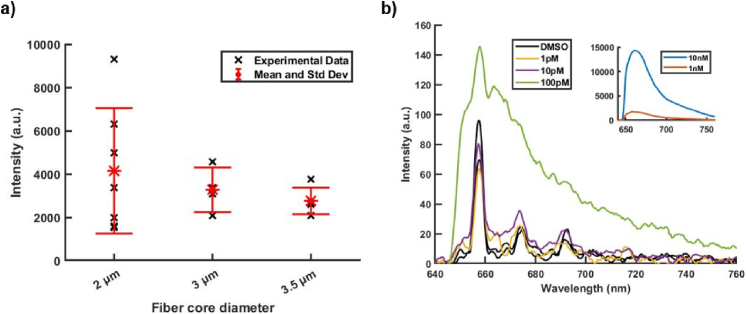
a) Fluorescence intensity, average value and standard deviation for 2 µm, 3 µm and 3.5 µm with a fiber length of 17 cm (Cy5 concentration = 10 nM). b) Fluorescence spectra of Cy5 including background signal of DMSO (Fiber length = 17 cm and laser power = 50 mA). The inset graph shows the dye concentration of 1 nM and 10 nm. LOD reach for 3.5 µm core size is 100 pM.

Fluorescence intensity were measured for various fiber core sizes within the same conditions (laser power, fiber length, and dye concentration are identical for all measurments). For each core size, 8 fibers samples were measured. The maximal intensity of each fluorescence spectrum are plotted in Fig. 5(a). with the mean values and the standard deviation. As expected from the results on the coupling efficiency, the measurement repetability is improved with the 3.5 µm core-size Sus-PCF. The standard deviation diminishes from 70% of the mean value for a 2 µm core size to 22.5% for a 3.5 µm core size. This trend is consistent with the numerical simulation of the coupling efficiency that the mean value is maximal for a core size of 3.5 µm. Despite the usual trend to use an optofluidic fiber with the smallest core size (≤ 2 µm), exhibiting the largest evanescent field [19,26,28], it is preferable to use of fiber with core size of 3.5 / 4 µm for real-life biosensing applications. The 3.5 µm core-size Sus-PCF offers higher light coupling efficiency and fluorescence measurement repeatability, easier light injection operability, and weaker sensitivity to system variations (coupling misalignment). Furthermore, the reduction of the fluorescence intensity due to lower evanescent field is rather modest, as the maximum intensity is only 1.5 lower than the one measured with the 2 µm core-size Sus-PCF. This could be explained by the larger coupling efficiency (of about 1.2 times) and a stronger attenuation (from the liquid absorption) of the light propagating in the 2 µm core-size Sus-PCF.

In order to assess the limit of detection (LOD) for Cy5 of the 3.5 µm core-size Sus-PCF, the fluorescence spectrum was measured for different concentration from 10 nM to 1 pM. A novel fibre sample was used for each measurement. Three measurements were realised for each concentration. A control measurement was realized by filling the fiber with only DMSO solvent. The results of this experiment are presented in Fig. 5. The fluorescence spectra of Cy5 with the intensity proportional to the dye concentration are obtained for the concentration of 10 nM, 1 nM and 100 pM. For lower concentration, the fluorescence spectrum overlaps with the background spectrum of the DMSO. The LOD is then determined to be 100 pM as it is the lowest concentration that can be detected without overlapping with the DMSO control spectrum. It is worth to notice that this level surpasses the LOD of 300 pM for fluorescein detected using Sus-PCF with a core size of 0.5 µm [38]. This outcome highlights the sensitivity of the 3.5 µm core fiber for fluorescence sensing. It is worth to notice, the fiber length could be increased for compensating the difference in sensitivity with the 2 µm core-size Sus-PCF. This strategy is nevertheless limited by the saturation effect appearing when the attenuation from the fiber (including liquid absorption) overcomes the fluorescence signal.

### 3.4 Detection of haptoglobin protein

**Fig. 6.**
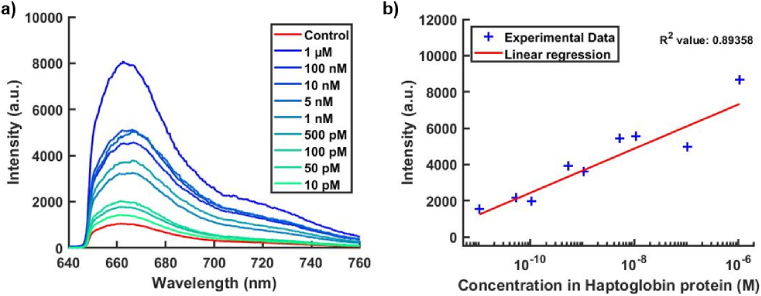
a) Average fluorescence spectra of Cy5-labelled antibody capture by specific binding with haptoglobin protein. Concentration of dye is constant for all measurement and signal intensity depends on the concentration of protein immobilized around the fiber core. (Core size = 3.5 µm, fiber length = 20 cm, laser power = 0.3 mW). b) Calibration curve showing the relation between fluorescence intensity and Haptoglobin protein concentration.

As a proof of concept, we used the 3.5 µm core size Sus-PCF for the detection of haptoglobin protein, a significant biomarker associated with various diseases and present in blood plasma. In Fig. 6(a), we showcase the average fluorescence spectra for various protein concentrations. Each measurement employs four fibers to obtain standard deviation values for each concentration. The control value corresponds to the immobilization of BSA protein, where the antibody lacks specific binding and is removed after washing. The residual signal may arise from active sites in the APTES layer not entirely blocked by the BSA. It is notable that all spectra using haptoglobin exhibit a higher fluorescence signal, affirming the capability of our protocol to attach the protein within the fiber. A calibration curve is plotted in Fig. 6(b), highlighting the linear relationship between protein concentration and fluorescence intensity. For this measurement, we calculated an R^2^ value of 0.89. Moreover, the average standard deviation values for each protein concentration were determined to be 40%, consistent with the repeatability achieved with this fiber.

We demonstrate the capability to detect haptoglobin protein concentrations down to the 10 pM level. This sensitivity places our approach in the same range as typical ELISA assay platforms, as reported in the literature [39]. These initial findings indicate that Sus-PCFs can achieve notable sensitivity with only a minimal sample volume, in contrast to the ELISA platform. Additionally, refining the fluorescence measurement protocol holds the potential to detect even lower protein concentrations, surpassing the performance of ELISA.

## 4. Conclusion

We demonstrated the realization of an optofluidic platform based on an optimized Sus-PCF offering higher light coupling efficiency and fluorescence measurement repeatability, easier light injection operability, fast liquid infiltrating into the fiber, and weaker sensitivity to system alignment variation. Fluorescence intensity of Cy5 dye is enhanced into the optofluidic fiber by an order of magnitude (in comparison to a standard cuvette). In contrast to theoretical expectations and current uses favoring smaller core size, we demonstrated that a core size of 3.5 µm /4 µm leads to a maximal coupling efficiency and reliability, improving the fluorescence measurement repeatability from a standard deviation of 70% (of the mean value) for a 2 µm core size to 22.5% for a 3.5 µm core size. This Sus-PCF enables the detection of Cy5 at concentrations as low as 100 pM, emphasizing an ultra-high sensitivity that is only 1.5 time lower than the 2 µm core size fiber. The results of this first study on the measurement reliability of fluorescence sensing with optofluidic fibers are pivotal for developing fiber-based platforms for biosensing applications. In this prospect, we have exploited the reliability of our platform by detecting haptoglobin protein, which is relevant biomarker in numerous diseases such as hemolytic anemia, certain cancers, and inflammatory disorders. To emphasis the simplicity of our optofluidic platform, haptoglobin detection was realized by using commercially available Cyanine5-labeled antibodies. The limit of detection of 10 pM illustrates furthermore the high sensitivity of our optofluidic platform. In this context, our PCF sensor can be simply adapted with different commercially available bio-recognition elements to sense various biomarkers (proteins, DNA, etc.) with higher sensitivity than current methods. Furthermore, PCF offers the versatility to be designed for any wavelength range. Recent studies have shown applications where biomarkers exhibit auto-fluorescence in the UV range where PCF can be used as a label-free sensor [11,40]. Additionally, our platform can be exploited for detecting biomarkers associated with specific diseases in a small amount of liquid sample, such as the detection of cancer biomarkers on exosomes. The potential of using this platform in a clinical environment could enable the direct detection of haptoglobin protein, at the hospital, by filling the fiber with the patient’s fluid (with minimal preparation), resulting in a reduced waiting time for diagnosis results.

## Data Availability

Data underlying the results presented in this paper are not publicly available at this time but may be obtained from the authors upon reasonable request.
